# Detection of red blood cell surface antigens by probe-triggered cell collision and flow retardation in an autonomous microfluidic system

**DOI:** 10.1038/s41598-017-01166-9

**Published:** 2017-04-21

**Authors:** Éva Sautner, Krisztián Papp, Eszter Holczer, Eszter L. Tóth, Rita Ungai-Salánki, Bálint Szabó, Péter Fürjes, József Prechl

**Affiliations:** 1 Budapest University of Technology and Economics, Budapest, 1111 Hungary; 2 MTA-ELTE Immunology Research Group, Budapest, 1117 Hungary; 3 Inst. of Technical Physics and Materials Science, Centre for Energy Research, HAS, Budapest, 1121 Hungary; 4 Faculty of Information Technology and Bionics, Pázmány Péter Catholic University, Budapest, 1083 Hungary; 5 CellSorter Company for Innovations, Budapest, 1037 Hungary; 6 Nanobiosensorics Group at Inst. of Technical Physics and Materials Science, Centre for Energy Research, HAS, Budapest, 1121 Hungary; 7 Department of Biological Physics, Eötvös Loránd University, Budapest, 1117 Hungary; 8 Diagnosticum Zrt., Budapest, 1047 Hungary

## Abstract

Microfluidic devices exploit combined physical, chemical and biological phenomena that could be unique in the sub-millimeter dimensions. The current goal of development of Point-of-Care (POC) medical devices is to extract the biomedical information from the blood. We examined the characteristics of blood flow in autonomous microfluidic devices with the aim to realize sensitive detection of interactions between particulate elements of the blood and the appropriately modified surfaces of the system. As a model experiment we demonstrated the fast analysis of the AB0 blood group system. We observed that the accumulation of red blood cells immobilized on the capillary wall leads to increased lateral movement of the flowing cells, resulting in the overall selective deceleration of the red blood cell flow column compared to the plasma fraction. We showed that by monitoring the flow rate characteristics in capillaries coated with blood type reagents it is possible to identify red blood cell types. Analysis of hydrodynamic effects governing blood flow by Finite Element Method based modelling supported our observations. Our proof-of-concept results point to a novel direction in blood analysis in autonomous microfluidic systems and also provide the basis for the construction of a simple quantitative device for blood group determination.

## Introduction

Medical applications of microfluidic systems hold the promise of miniaturization and accelerated testing for biomarkers or cells. While classical assays of molecular biology and immunology can be scaled down for these systems, it is also of high interest to introduce novel principles taking advantage of the reduced dimensions and distinct physical phenomena observable on this scale. Since cells’ dimensions are in the low micrometer range it seems intuitive to develop applications that utilize cells as components of the assay. We attempted to combine a self-driven microfluidic system and cell-molecule interactions in order to generate a simple but robust assay and readout system for Point-of-Care (POC) diagnostics. Here we provide proof-of-principle of the system using the human blood group markers as a model.

Self-driven microfluidic devices are ideal for POC testing as they require no external power supply and are less prone to user-introduced error. Autonomous microfluidic devices exploit capillary forces for transferring liquid in the system^1^. Key factors affecting the performance of these devices are structure/geometry and material composition. Capillary forces depend on the interactions of the fluid, the gas phase and the capillary wall. Geometries modulate the contact angle of the proceeding liquid front thereby affecting liquid movement. Based on this knowledge complex systems with different functional units can be designed^2^.

**Figure 1:**
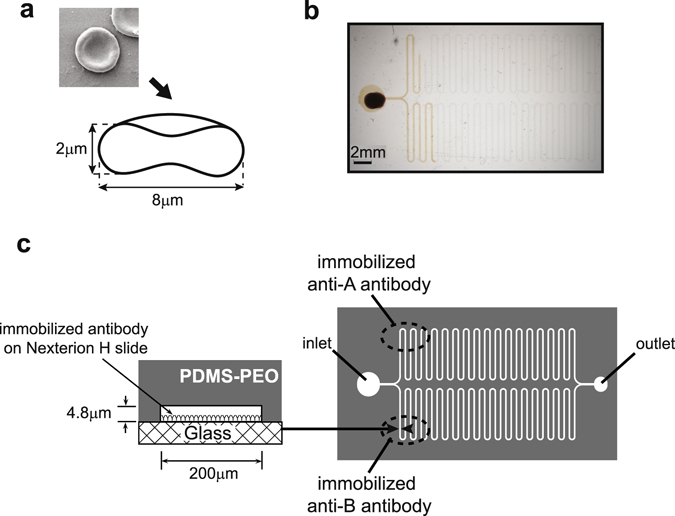
RBC dimensions and design of microfluidic chamber. (**a**) A scanning electron microscopic picture and a cross-sectional view of a red blood cell that indicates it’s shape and average dimensions. (**b**) The photo shows the capillary system filled by blood. (**c**) The microfluidic device was formed by aligned attachment of PDMS-PEO based flow cell containing microfluidic channels onto slides functionalized by anti-A and anti-B reagents in the indicated regions. The left panel presents the cross-sectional view of the winding channel.

Blood is a fluid tissue comprising particulate elements and dissolved molecules. It is in fact the most often sampled human tissue because of relatively easy availability and because of the wealth of information it carries. Of the particulate elements red blood cells (RBC) are the most abundant with numbers in the range of 4 to 5 million cells per microliter. This accounts for approximately 50% of the blood volume and an enormous surface area owing to the biconcave disc shape of the cells. The diameter of a RBC is 7.5–8.5 μm, with a width of 1.7–2.2 μm in the ring^3^ and 0.5–1 μm at the centre (Fig. 1a). The cell has a number of molecules embedded in the membrane; some of these are receptors for circulating ligands in the blood. RBCs carry oxygen bound to hemoglobin, in case of sever loss they need to be replaced to sustain this capacity. It has long been known that RBC transfusion is only possible between matched donors and acceptors, depending on their blood type. Blood type is a genetically determined property that appears as the quality of glycoproteins on RBCs and also as the immunological recognition of these glycoproteins^4, 5^. In the case of AB0 blood typing group system RBC display A and/or B type antigen on the surface and antibodies are present in the plasma against the non-expressed antigen (Table 1).

**Table 1:** Characteristics of the AB0 blood groups.

Blood group	0	A	B	AB
RBC antigens	—	A	B	A & B
Serum antibodies	anti -A & B antibody	anti-B antibody	anti-A antibody	—

## Results

### Generation of a microfluidic system for testing RBC in whole blood

The general aim of our experiments was to identify microfluidic structures and materials suitable for the detection of durable interactions between blood cells and the walls of the autonomous device. To this end we designed and tested microfluidic structures with transparent walls, made of hydrophilic surfaces and with dimensions comparable to that of RBC (Fig. 1). We assumed that by using microfluidic channels with restricted height but using relative large width, binding of the cells to the wall would lead to gradually increased hydrodynamic drag. We chose to use a height, which does not allow a “standing” RBC to pass through, increasing the frequency of contacts between the cells and the wall. On the other hand, in order to allow the passage of RBC even when a layer is formed from bound cells, height was chosen to be comparable to twice the breadth of RBC. Winding channels allowed for the observation of long flow paths over a relatively short distance on the chip. The device shown in Fig. 1 thus drives the movement of whole blood by capillary forces through a bifurcation leading into two identical winding channels, which are coated with distinct reagents in our experimental setup.

### Positive reaction results in RBC retardation and plasma separation

**Figure 2:**
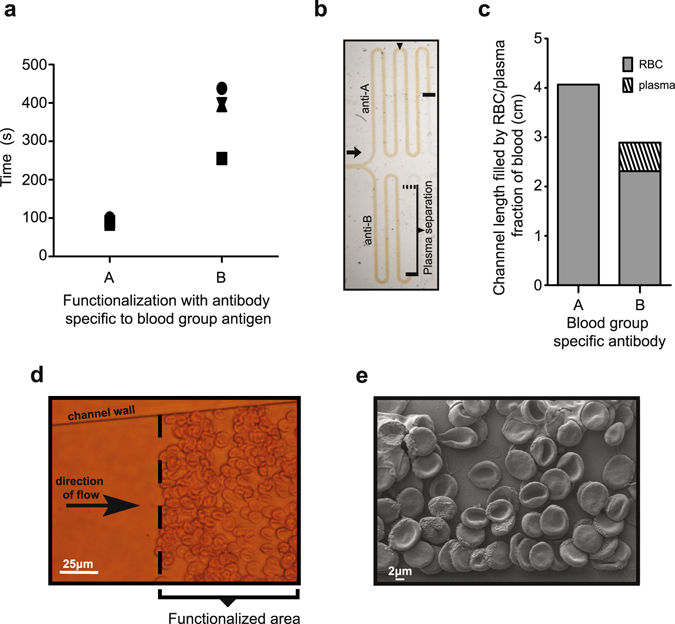
RBC retardation and plasma separation. (**a**) Graph shows the time that needed for a blood group B blood to reach the second loop (indicated by arrowhead in panel b) in the two arms functionalized by anti-A or anti-B group specific antibodies. The experiment was repeated 4 times, data pairs of the same run are represented by identical symbols. (**b**) A picture taken at the end of experiment (7 min) shows that the plasma separated from red blood cells only in the arm that was functionalized by anti-B antibodies. (**c**) Graph presents the length of RBC or plasma of a blood group B blood in the two arms at the end of experiment. (**d**) Light microscopic and (**e**) SEM pictures shows that RBC’s binds to the functionalized area.

Upon introduction into the inlet port blood autonomously started to fill up the device, proceeding into the test channels with functionalized surface. One arm was functionalized with anti-A, the other with anti-B reagent (Fig. 1c). When group B blood filled the capillary system then the velocity of flow was dissimilar in the two arms: in the anti-B reagent coated arm the blood needed more time to reach the second loop following the functionalized area (Fig. 2a). Experimental observation was stopped when capillary forces were counterbalanced by hydrodynamic drag and flow of blood was no longer visible in either of the channels. By the end of the experiment, around the 7th minute, the moving front of the plasma and the RBC separated in the arm with positive reaction as the plasma reached a longer distance than the RBC (Fig. 2b,c). RBCs bearing the relevant antigens were captured by the reagents in a specific manner (Fig. 2d,e) resulting in the generation of a layer of RBC bound to the functionalized surface.

### Captured RBC in the functionalized area form obstacles

**Figure 3:**
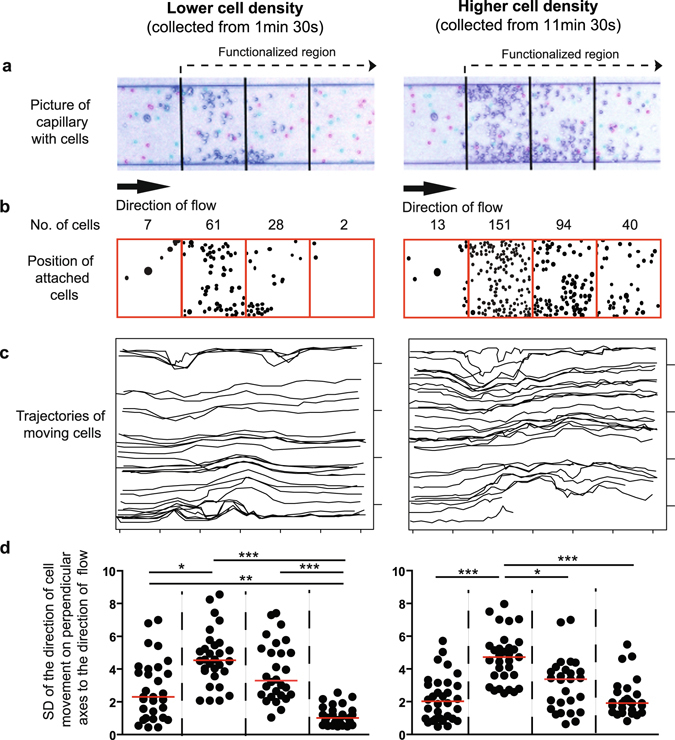
Attached cells in the trapping region perturb the motion of moving cells and force them perpendicular directions to the flow. Routs of moving RBC’s were analysed in the capture region in case of two different bound cell density (right and left panel). (**a**) Two microscopic pictures about the capillary taken in delayed time points (pink and light blue colours) were overlaid to visualize the attached cells (dark blue). Field of vision was divided to four equal regions those were analysed separately, the 2–4 regions were functionalized as indicated. (**b**) Positions of bound cells were indicated by black dots for better visualization. (**c**) Trajectories of 30–30 moving RBC’s were analysed. (**d**) Graph shows the SD of movement of cells in perpendicular direction to the flow of blood. Friedman and Dunn’s statistical tests were applied to reveal significant differences (*p < 0.05; **p < 0.01; ***p < 0.001, n = 30).

To better understand the phenomenon that occurs in the functionalized area we recorded the trajectories of individual moving RBC. During the assay the capture of passing RBC continued, resulting in a gradually increasing area covered by stationary RBCs. By tracking RBC trajectories we found a significant increase in lateral movement due to collision of moving cells with stationary cells (Fig. 3).

### Simulation of blood flow

**Figure 4:**
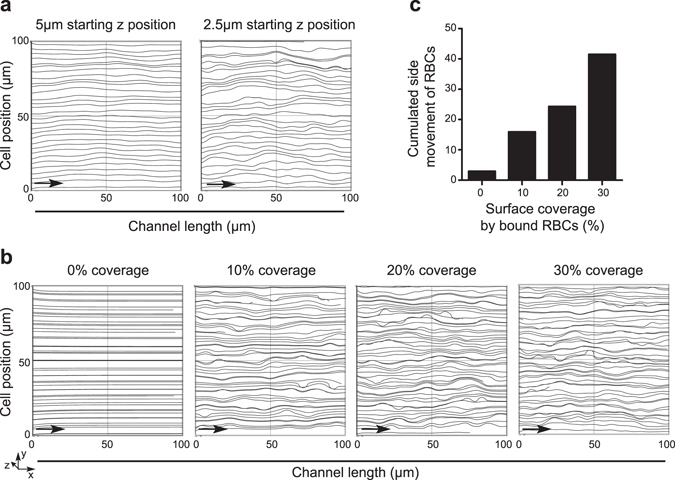
(**a**) Lateral projection of the FEM modelled RBC trajectories over the trapped cells in case of 5 μm and 2.5 μm starting z position. Arrows indicate the direction of simulated flow. (**b**) Simulated particle trajectories over the functionalized area of the microfluidic system in case of different trapped cell coverage on the surface of the 100 × 100 μm^2^ area. (**c**) Cumulated side movement (in y and z directions) of the RBCs crossing the functionalized area covered by bound cells (FEM model).

To confirm that the observed increased frequency of collisions can lead to a decreased effective flow rate of RBC we used computer modelling, testing effects of channel height and surface coverage by stationary randomly distributed non-overlapping RBCs. As Fig. 4a presents the trajectories of the simulated RBCs are not affected by the stationary cells in case the starting z coordinate of the particle is close to or higher than 5 μm. Thus the efficiency of the microfluidic assay is quite sensitive for the channel height and the effective hydrodynamic impact of the captured cells is limited to the vicinity of the functionalized surface. Modelling confirmed our assumption that the channel height should be comparable with twice thickness of the RBCs (cca. 5 μm) to achieve significant trajectory modification without clogging the channels. The trajectories of the RBCs over the functionalized area were also analysed by Finite Element Method (FEM) simulation in order to reveal the retardation effect caused by the captured RBCs. Channel height was defined as 5 μm according to the preliminary channel height analysis, and the starting z coordinate of the monitored 30 particles was set to 2 μm, monitoring cell movement over the area with different trapped cell densities (Fig. 4a–c). Higher trapped cell numbers generated more intensive lateral movements of the travelling particles causing the decrease of the effective velocity along the channel (dimension x).

### Plasma separation occurs over a wide range of RBC densities

**Figure 5:**
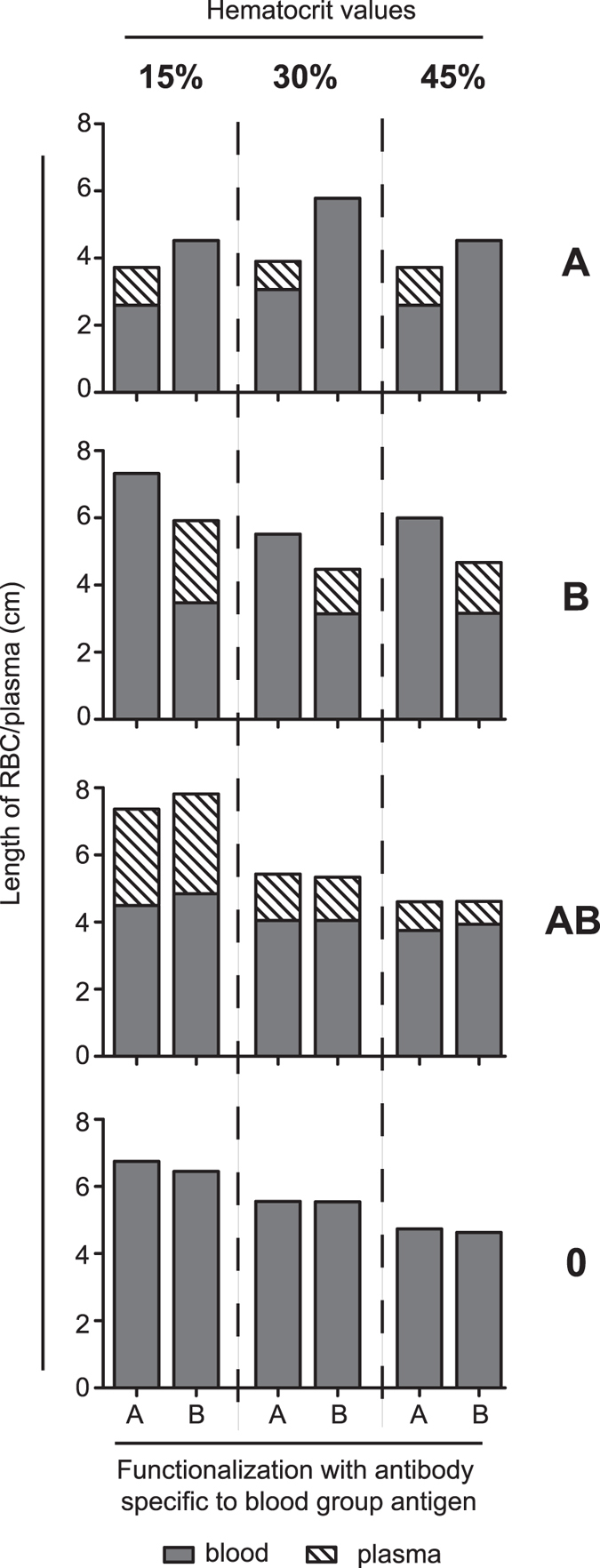
Effect of various hematocrit values on blood group determination. The hematocrit of blood was artificially set to the three indicated value in case of blood samples from the four different blood groups (A, B, AB, 0) then tested in microfluidic channel system where the two arms were functionalized by anti-A or anti-B group antibodies. The length of RBC and separated plasma regions are indicated by grey or striped columns. Graph shows representative results from three replicates.

A significant volume of whole blood is contributed by the particulate elements, especially by the RBC, which constitute between 30 to 50% of the volume. The medical term for this percentage is hematocrit, its value depends on gender, age and physical condition. Since the rate of RBC capture by the functionalized surface could be influenced by hematocrit values we tested a range of adjusted RBC concentrations to establish the validity of our assay for determining blood group AB0 (Fig. 5). The phenomenon of RBC retardation and plasma separation was observed with physiological and sub-physiological hematocrit values. The use of 60% RBC did not generate reliable results, presumably due to increased viscosity and fast drying and clogging in the inlet port. Importantly, all four different AB0 blood groups showed similar behaviour in our assay (Fig. 5). In the case of blood group 0 the rate of flow is comparable in the two channels with no plasma separation, while for blood group AB plasma separation occurs in both channels.

## Discussion

Blood groups are genetically determined self-antigenic variations presenting as immunologically detectable molecular differences on RBC. Genetic testing can determine variability in genes that define molecular properties of antigens displayed by RBC^6^ and also by other cell types. Serological tests complement this information by determining the phenotype of circulating cells (forward typing) and reactivity of serum antibodies (reverse typing). Antibodies binding to RBC antigens can mediate agglutination and lysis of target cells, which serve as simple and robust methods of typing. Whether on slide, in tubes, microtiter plates, gels^7^ or in microfluidic systems, most current approaches^8^ of serological blood typing use the phenomenon of agglutination. Inherently these approaches require the mixing of the blood sample with the agglutinating antibody. A paper-based microfluidic approach by Noiphung *et al*.^9^ exploits aggregation-mediated changes in the flow, resulting in a system with simple visual readout. Kline *et al*. also utilized aggregation phenomena, though on a microscopic scale, to identify AB0 blood types^10^ in a two-phase microfluidic system and microscopy to characterize aggregation in droplets. Quantitative assessment of agglutination can help the identification of subtypes with varying antigen densities^11^. Technologies using surface coating and monitoring interactions of RBC with the functionalized surface require specialized instruments for readout, such as surface plasmon resonance^12, 13^ detection. The approach we present here is based on device surface-RBC interactions, in a simple microfluidic device, yet with an easily quantifiable readout (Table 2).

**Table 2:** Comparison of widely used agglutination methods (slide, tube microplate or column/gel filtration format)^8^ and the presented flow retardation microfluidic assay.

	Agglutination methods in general	Flow retardation microfluidic assay
Blood required	>10 µl	3 µl
Assay duration	>10 min	7 min
Quantitation	only with instrument	manually possible
Price	variable	>slide
Reverse typing	possible	not possible
Sample preparation	pipetting/mixing/centrifugation etc.	no preanalytical steps

The separation of blood plasma from RBC by gravity in static state is known as erythrocyte sedimentation rate. RBC sediment due to their higher density, rate is influenced by composition of plasma and the formation of stacks or rouleau of RBC. Aggregates of RBC also tend to form in diluted blood flowing in microfluidic devices, especially at low shear rate^14^. Further reduction of the microfluidic channel height resulted in plasma separation in our hands as well. While slower rate of flow may contribute to this phenomenon our results suggest that more frequent collisions of RBC with channel walls could also result in selective slowing down of cells. Upon passing through the region with immobilized antibodies there is a net reduction of RBC numbers in the fluid phase. Therefore we cannot rule out that this loss may also contribute to the development of a RBC-free plasma phase but the relatively delayed appearance of plasma separation points towards the collision-mediated scenario. Ultimately these events lead to a gradually increasing lag of the RBC front behind the plasma front.

The microfluidic system we present here has several potential advantages as a point-of-care device. It is a self-driven system, no pumps or external driving force is needed. Reagents are coated in the microfluidic test channels, no mixing of sample and reagent is required. The microfluidic device is closed except for the inlet and outlet ports, protecting the reaction from environmental interference. The system can be multiplexed by introducing more test channels with different reagents. There is only one inlet port for combined determination of reactivities, no preanalytical steps - such as dilution of blood - are required, a drop of blood can be directly introduced into the system. Anti-coagulation agents could be adsorbed in the inlet port. In addition to simply reading the results by the naked eye, quantitative results can also be generated and digitally archived by supplementing the device with simple optical sensors detecting RBC movement.

The sensitivity of the technology to differences in surface antigen density of RBC remains to be determined. Subtyping of type A blood to A1 and A2 can pose a challenge in this respect. The incorporation of positive and negative control channels with reagents against universal antigens and with no RBC reactivity, respectively, can further enhance quantitative measurements. We assume that the phenomenon of probe-triggered cell-collision and flow retardation can be exploited for the development of a range of other assays that utilize simple autonomous microfluidic systems and blood for medical diagnostics. In the special case of the AB0 blood group system, the use of two channels is just suitable to identify all four possible phenotypes: AB, A, B and 0. The system can have as many channels as required, as long as parallel flow can be maintained in the channels. Unlike a pump-driven system, where the blockade of one of many parallel channels will result in an increased flow in the free channels, our autonomous system is driven by negative capillary pressure. Here the blockade of one channel/arm will not affect flow in the other channels.

In summary we have identified and optimized an autonomous microfluidic system suitable to promote strong interactions between RBCs and functionalized microfluidic channel surface resulting in altered flow properties of the tested blood sample. This alteration is characterized by gradual increase of the frequency of collisions between moving and captured cells, a concomitant gradually increasing lateral displacement of cells leading to decreased effective velocity, an overall retardation of blood flow and a concomitant and more pronounced retardation of the RBC component of blood. Using the model system of blood type group AB0 we also demonstrated that this phenomenon can be applied to reliably identify the blood group with absolutely no preanalytical steps.

## Methods

### Fabrication of microfluidic chambers

Our hypothesis implied that the binding of particulate elements of blood to the walls of a microfluidic system would result in retardation of the fluid motion. In order to be able to observe fluid movement as long as possible on the smallest possible footprint we designed a bifurcated winding channel system (Fig. 1b,c). Blood sample is introduced via an inlet port, enters a common capillary channel (300 μm width), and then it is diverted into two channels of identical structure (200 μm width). Microfluidic chambers were fabricated by soft lithography technique using SU-8 pattern as moulding replica. For the replica SU-8 3005 or 3010 (MicroChem) photoresist was spin coated at 4000 rpm onto a silicon wafer using Brewer Science 200CBX spincoater. UV-lithography technique was used to pattern the photoresist. The photomask for the lithography process was made by a Heidelberg DWL 66+ laser pattern generator. A Süss Mikrotech MA6 mask aligner was used to expose the SU-8 layer, then the resist was developed in 1-methoxy-2-propyl acetate solution. Post-exposure bake was applied on the wafers at 90 °C in the Amending the hydrophobic polydimethylsiloxane (PDMS, Sylgard 184 - Dow Corning) with dimethylsiloxane ethylene oxide block copolymer (PEO - Sigma-Aldrich) results the modified polydimethylsiloxane (PDMS-PEO) with hydrophilic surface properties where autonomous flow of blood can be achieved. The concentration of PEO molecules is critical for the assay, considering that lower concentrations caused insufficient capillary transfer and higher concentrations significantly deteriorated the physical (optical, mechanical) properties of the polymer. The microfluidic chambers were made in PDMS-PEO by mixing Sylgard 184 elastomer and cross-linking agent with PEO in 100:10:0.75 volume ratio. The mixture was cured on the SU-8 replica for 1 h at 80 °C, then the cross-linked polymer was peeled off from the master.

### Preparation of capillary system

Hydrogel coated glass slides (Nexterion H slides, Schott) were functionalized by pipetting 3 μl anti-A or anti-B blood type specific reagents (Diagast) to the indicated regions (Fig. 1c), then incubated at 37 °C for 1 h. Unbound reactive groups were blocked by bathing in 100 mM TrisHCl. Following 3 × 5 min washing with PBS, slides were rinsed in water then dried. The PDMS microfluidic blocks were attached onto functionalized slides to form the capillary system simply by gently pressing the two parts together. There was no need for extra bonding process, the device being a capillary force-driven low pressure system.

### Detection of blood flow

RBCs with known AB0 group properties were ordered from Bio-Rad (Biotestcell). Blood of volunteers from among the authors of this paper was obtained by finger prick and mixed with 0.5 M EDTA to reach 20 mM final EDTA concentration that prevents blood coagulation. Other types of anticoagulants (heparin, hirudin, sodium citrate) were also successfully used in our assay (data not shown). Hematocrit values were set by the appropriate mixture of separated RBC and plasma fractions. Unless stated otherwise, 3 μl blood was added to the inlet of the device and the flow was recorded for a maximum of 7 min by Leica DMS1000 microscope. For the measurement of plasma separation, the foremost RBC that was followed by a continuity of RBCs was regarded as the RBC-plasma border, while the plasma-air junction in the centre of the channel was regarded as plasma front. The distance between these two points was calculated along the centre of the channel using a digital image with a scale bar. For visualization of functionalized area-bound cells, PBS was used to wash away the unbound cells and a picture was taken by Olympus IX 70 microscope. Following subsequent fixation with 4% paraformaldehyde and sputtering 5 nm thick gold layer onto the cell plate the lateral/spatial distribution of the captured RBCs in the microfluidic channel was visualised by Zeiss LEO 1540XB FESEM scanning electron microscope using 2 keV acceleration voltage. To follow the route of single RBC’s in capture area that was coated by anti-A antibodies, 50 times diluted blood group A blood in PBS was added to the capillary system. The video of cell movement was taken for 12 minutes by a Zeiss Axio Observer A1 microscope equipped with an Andor Zyla 5.5 camera and only the beginning (with yet lower attached cell density) and ending (with higher attached cell density) part of the video was analysed. Ffmpeg software was used to convert frames of video to separated pictures then Manual Tracking plugin of ImageJ software was applied to follow trajectories of 30–30 RBC’s.

### FEM simulation of the blood flow

The hydrodynamic effects governed by the interaction evolving between the freely moving and the trapped RBCs were modelled by COMSOL Multiphysics code utilizing its CFD and particle tracing modules. The particle movement was calculated after solving the flow velocity field described by Navier-Stokes equation utilizing the Stokes equation. Trapped RBCs were modelled by geometric obstacles in the microchannel. Randomization of these obstacles was implemented in a MATLAB script and imported into COMSOL Multiphysics. The generated lateral distribution of the RBCs was compared to the experimental results recorded by SEM (see Supplementary Fig. S1).

Laminar flow was considered with normal inflow velocity (0.05 μl/s) as inlet, pressure (reference value) as outlet, and no slip as wall boundary conditions. To aid convergence 0.05 μl/s was set for x component of the velocity field as initial value. Particle properties were set to match RBC properties (density: 1030 kg/m^3^, diameter: 4 μm). Particles were released from a line with uniform distribution across the channel. Wall boundary condition was set to “Stick” and outlet was set to “freeze”. The flow velocity field was considered stationary, while the particle trajectories were calculated with time dependent solver. Numerical results were exported and further analysed with a dynamic Matlab script to calculate the sum of relative lateral displacements of the particles along the channel.

Numerical simulations are based on a pre-calculated stationary velocity field as a result of solving the Navier-Stokes equation (equation 1) numerically with finite element method.

1 $$\frac{\partial \underline{v}}{\partial t}+(\underline{v}\cdot \nabla )\underline{v}=-\frac{1}{\rho }\nabla p+v{\nabla }^{2}\underline{v}$$

Where v is the velocity (m/s), p is the pressure (Pa), ρ is the density (kg/m^3^) and ν is the kinematic viscosity (m^2^/s) of the fluid.

The particle tracing based model calculates, follows and depicts the individual particle trajectories according to the hydrodynamic drag force described by Stokes’ law (equation 2).

2 $$\frac{d}{dt}({m}_{p}{\underline{v}}_{p})=3\pi \mu {d}_{p}(\underline{v}-{\underline{v}}_{p})$$

Where m_p_ is the mass of the particle (kg), v_p_ is the velocity of the particle (m/s), μ is the dynamic viscosity of the fluid (Pa·s), d_p_ is the particle diameter (m) and v is the local velocity vector of the fluid (m/s).

The cumulated side movement of RBCs in Y and Z directions during crossing the functionalized surface among the bound cells, calculated from the trajectory model (equation 3).

3 $$Q=\sum _{n=1}^{N}\frac{\sqrt{{\rm{\Delta }}{y}_{n}^{2}+{\rm{\Delta }}{z}_{n}^{2}}}{{\rm{\Delta }}x},$$

Where ∆x = L/N is the movement of the particle during a timestep of the simulation along the *L* long modelled part of the channel. ∆y_n_ = y_n_ − y_n−1_ and ∆z_n_ = z_n_ − z_n−1_ are the particle coordinate change between two timestep in the given direction, representing the off-directional movement of the cells.

## Electronic supplementary material

Supplementary Information
