# Supplementary material for: Detection of red blood cell surface antigens by probe-triggered cell collision and flow retardation in an autonomous microfluidic system
Source: Sci Rep. 2017 Apr 21;7:1008. doi: 10.1038/s41598-017-01166-9 (PMC5430922; doi:10.1038/s41598-017-01166-9)
Supplement: Supplementary file 1 — Supplementary Information [file 41598_2017_1166_MOESM1_ESM.pdf]

Detection of red blood cell surface antigens by probe-triggered cell collision and flow retardation in an autonomous microfluidic system

Éva Sautner, Krisztián Papp, Eszter Holczer, Eszter L. Tóth, Rita Ungai-Salánki, Bálint Szabó, Péter Fürjes and József Prechl

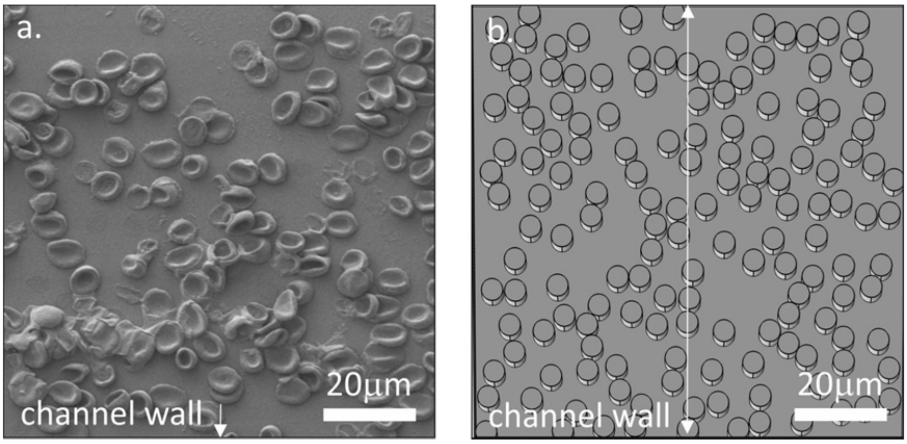

Supplementary Figure S1. Lateral distribution of the bound RBCs in the microfluidic channel (a) and the initial channel geometry of the FEM simulation (b) containing the randomly distributed model particles (in case of 30% surface coverage).
